# Sigma-1 receptor agonist PRE084 is protective against mutant huntingtin-induced cell degeneration: involvement of calpastatin and the NF-*κ*B pathway

**DOI:** 10.1038/cddis.2013.170

**Published:** 2013-05-23

**Authors:** A Hyrskyluoto, I Pulli, K Törnqvist, T Huu Ho, L Korhonen, D Lindholm

**Affiliations:** 1 Institute of Biomedicine/Biochemistry and Developmental Biology, University of Helsinki, Biomedicum Helsinki, Haartmaninkatu 8, 00014 Helsinki, Finland; 2 Minerva Medical Research Institute, Biomedicum Helsinki, Tukholmankatu 8, 00290 Helsinki, Finland; 3 Department of Biosciences, Åbo Akademi University, 20520 Turku, Finland; 4 Division of Child Psychiatry, Helsinki University Central Hospital, 00029 HUS Helsinki, Finland

**Keywords:** Sigma-1 receptor, NF-*κ*B, calpastatin, oxidative stress, HD

## Abstract

Alterations in mitochondria and increased oxidative stress are associated with the disease progression in Huntington's disease (HD). Endoplasmic reticulum (ER) stress and oxidative damage are linked through the close communication between the ER and mitochondria. Sigma-1 receptor (Sig-1R) is a chaperone protein in the ER that is involved in ER stress regulation, but little is known about its role in HD or the mechanisms for cell protection. Here we show that the Sig-1R agonist, PRE084 increases cell survival and counteracts the deleterious effects caused by N-terminal mutant huntingtin proteins in neuronal PC6.3 cells. Particularly, PRE084 increased the levels of cellular antioxidants by activating the NF-*κ*B pathway that is compromised by the expression of mutant huntingtin proteins. These results show that the Sig-1R agonist has beneficial effects in models of HD and that compounds affecting the Sig-1R may be promising targets for future drug development in HD.

Huntington's disease (HD) is an autosomal, dominantly inherited neurodegenerative disorder that is caused by an expansion of cytosine–adenine–guanine (CAG) repeats in the first exon of the huntingtin gene *IT15*, which encodes the huntingtin protein.^1^ The expansion of CAG repeats in huntingtin leads to the formation of neuronal intracellular and intranuclear aggregates.^2^ HD is characterized by severe motor and cognitive symptoms due to brain atrophy with loss of neurons especially in the striatum and cerebral cortex. The wild-type huntingtin protein (350 kDa) is expressed in most tissues and participates in protein trafficking, postsynaptic signaling, vesicle transport, transcriptional regulation, and in regulation of cell death. The precise molecular mechanisms by which mutant huntingtin induces nerve cell degeneration are not completely understood,^3^ but both loss-of-function and gain-of-function mechanisms have been described in HD.^4^ The mutant protein is cleaved to form N-terminal fragments containing the first 100–150 residues including the polyglutamine repeats that are thought to be the toxic species found in aggregates.^5^ Polyglutamine-expanded proteins cause disruption of many intracellular pathways including those in the endoplasmic reticulum (ER) leading to ER stress.^6^ ER stress triggers the unfolded protein response (UPR) that is mediated by three proximal ER sensors: PERK, ATF6, and IRE1. The UPR is a cellular adaptation mechanism but prolonged or severe ER stress can also activate signaling pathways leading to apoptosis.^7^ ER stress and oxidative damage are linked through the close communication between the ER and mitochondria. Oxidative stress can lead to a toxic increase in reactive oxygen species (ROS) production, which causes damage to cell membranes and aborts normal cellular functions.^3, 8^ Previous studies have shown that oxidative stress has a role in the neurodegenerative processes in HD.^3, 9^

The Sigma receptors are non-opioid receptor proteins^10^ that are classified into two major subtypes; Sigma-1 and Sigma-2 receptors.^11^ The Sigma-1 receptor (Sig-1R) has been cloned^12^ but the sequence of the Sigma-2 receptor is so far not known. Hayashi and Su^13^ have shown that Sig-1R is a chaperone protein located at the ER that specifically targets the mitochondria-associated ER membrane (MAM). Sig-1R is thought to form a Ca^2+^-sensitive chaperone complex with Bip prolonging Ca^2+^ signaling from ER into mitochondria by stabilizing the inositol 1,4,5-trisphosphate receptor (IP3R). Sig-1R is upregulated by ER stress and overexpression of Sig-1R regulates UPR signaling.^13^ It is also known that Sig-1Rs can translocate from MAM to other parts of the ER and this event may be provoked by ER stress. Knockdown of Sig-1R is shown to promote apoptosis induced by ER stress and by ROS.^13, 14^

The function of Sig-1R has also been linked to neurodegenerative disorders as shown by analyses of materials from patients afflicted by these diseases. Thus, a postmortem study reported that Sig-1Rs were reduced in the hippocampus in Alzheimer's disease (AD)^15^ and Sig-1R levels were also decreased in specimens from patients with early Parkinson's disease.^16^ A recent study reported a mutation in Sig-1R in patients with juvenile amyotrophic lateral sclerosis (ALS),^17^ however little is so far known about the role of Sig-1R in HD.

In this work, we have studied the role of Sig-1R and its agonist 2-(4-morpholinoethyl)-1-phenylcyclohexane-1-carboxylate hydrochloride (PRE084) in a cellular model of HD with overexpression of wild-type and mutant huntingtin proteins in neuronal PC6.3 cells. We show that Sig-1R protein levels were decreased in mutant huntingtin-expressing cells and that PRE084 can restore the levels. PRE084 also protected PC6.3 cells from the deleterious effects of mutant huntingtin proteins via the upregulation of NF-*κ*B-p65 resulting in increased levels of cellular antioxidants and decreased oxidative stress. Increased calpastatin levels were crucial for the beneficial effects by PRE084 and Sig-1R overexpression whereas changes in mitochondrial calcium were not obvious in mutant huntingtin-expressing cells.

## Results

### Expression of Sig-1R protein is decreased in mutant huntingtin protein-expressing cells

**Figure 1:**
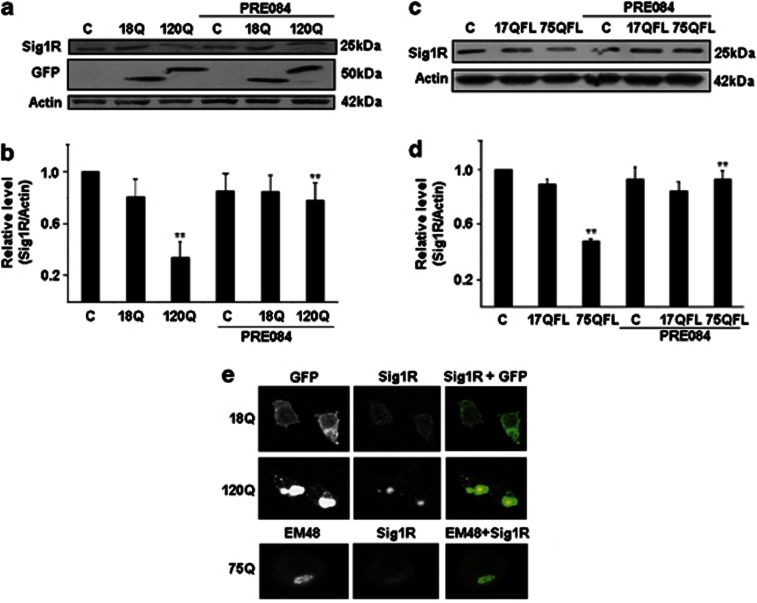
Levels of Sig-1Rs in PC6.3 cells expressing mutant huntingtin proteins. (**a**) Immunoblot. Cells were transfected for 24 h with expression constructs encoding different N-terminal huntingtin fragment proteins with 18Q and 120Q repeats. The compound 0.3 *μ*M PRE084 was added as indicated. The levels of Sig-1R were analyzed as described in the Materials and Methods. Sig-1R was decreased in cells expressing 120Q-huntingtin and PRE084 restored this level. *β*-actin was used as control. Expression of the GFP-huntingtin constructs is also shown. (**b**) Quantification was done using ImageJ. Values represent means±S.D., *n*=3. **P<0.01 for 120Q *versus* C and for PRE084+120Q *versus* 120Q. (**c** and **d**) Immunoblot and quantification. Cells were transfected for 48 h with FL huntingtin constructs having either 17 (17Q) or 75 polyglutamine (75Q) repeats. Sig-1R protein levels were decreased in cells expressing the disease-causing 75Q-huntingtin protein and restored by treatment with 0.3 *μ*M PRE084. *β*-actin was used as control. Quantification was done as above. Values represent means ±S.D., *n*=3. **P<0.01 for 75Q *versus* C and for PRE084+75Q *versus* 75Q. (**e**) Confocal microscopy. Immunofluorescence (green) of PC6.3 cells transfected for 24 h with huntingtin fragment proteins containing 18Q (upper panels) and 120Q repeats (middle panels). Immunostaining was done using specific antibody against Sig-1R (red fluorescence). Sig-1R is present in part in cytoplasmic aggregates in cells expressing120Q-huntingtin in the merged picture (yellow color, arrow). Scale bar, 15 *μ*m. Lower panels, immunostaining using the EM48 antibody recognizing mutant huntingtin aggregates. Note a partial co-localization with Sig-1R

Using neuronal PC6.3 cells, we found that the protein levels of Sig-1R were decreased in cells expressing N-terminal huntingtin fragment proteins having 120 polyglutamine repeats (120Q-huntigtin) (Figures 1a and b). However, cells expressing the 18Q-huntingtin-fragment protein exhibited Sig-1R levels similar to those in controls (Figure 1a). Cells expressing the full-length diseases- huntingtin protein (75QFL) had reduced levels of Sig-1R levels (Figures 1c and d), whereas cells overexpressing wild-type 17Q-huntingtin (17QFL) were not different from controls (Figures 1c and d). Stimulation of the PC6.3 cells with the Sig-1R agonist PRE084 elevated the Sig-1R levels reduced by mutant huntingtin proteins (Figures 1b and d). To study whether the changes in Sig-1R occurred at the RNA levels, we performed quantitative PCR (qPCR) that showed no significant alterations in Sig-1R mRNA (Supplementary Figure 1). This suggests that the regulation of Sig-1R by its agonist occurs at the post-transcriptional level. As shown using confocal microscopy, there was a co-localization of Sig-1Rs and the 120Q-huntingtin-fragment protein within the cytoplasmic aggregates in the PC6.3 cells (arrow in Figure 1e). This was not observed in PC6.3 cells expressing the 18Q-huntingtin-fragment protein (Figure 1e). The reason for this is not clear but it is known that Sig-1R has a function as a chaperone and might bind to protein aggregates.

### The Sig-1R agonist PRE084 counteracts cell degeneration and caspase activation induced by N-terminal mutant huntingtin proteins

**Figure 2:**
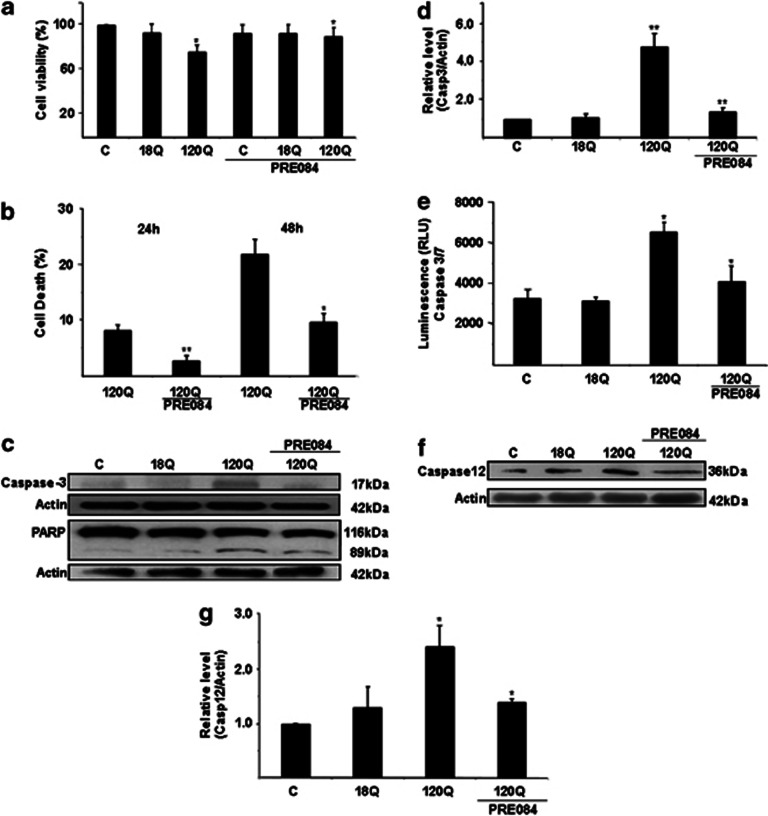
Stimulation with PRE084 counteracts the deleterious effects caused by N-terminal mutant huntingtin proteins. PC6.2 cells expressing 18Q- and 120Q-containing N-terminal huntingtin fragment proteins were left untreated or treated with the Sig-1R agonist, PRE084 as described in the Materials and Methods. (**a**) Cell viability was assessed after 24 h by the MTT assays as described in the Materials and Methods. PRE084 reduced cell death induced by the 120Q mutant huntingtin. Values represent means±S.D., *n*=3. *P<0.05 for 120Q *versus* C and for PRE084+120Q *versus* 120Q. (**b**) Cell death was assessed by counting number of apoptotic nuclei in 18Q and 120Q-huntingtin-fragment protein-expressing cells incubated for 24 h and 48 h without or with 0.3 *μ*M PRE084. Cell death increased in cells transfected with the 120Q huntingtin and this was reduced by PRE084. Values represent means±S.D., *n*=3. **P<0.01 for 120Q *versus* 18Q at 24 h and *P<0.05 at 48 h. (**c** and **d**) Immunoblot and quantification. Expression of 120Q-huntingtin-induced cleaved caspase-3 (17 kDa band) and this was reduced by addition of 0.3 *μ*M PRE084. The cleavage of PARP (89 kDa band) a downstream substrate of caspase-3 was also decreased by PRE084. *β*-actin was used as control. Values represent means±S.D., *n*=3. **P< 0.01 for 120Q *versus* C and for PRE084+120Q *versus* 120Q. (**e**) Activity of caspase-3 was measured as described in the Materials and methods. Note an increase in cells expressing 120Q mutant huntingtin that as reduced by 0.3 *μ*M PRE084. Values represent means±S.D., *n*=3. **P<0.01 for 120Q *versus* C and for PRE084+120Q *versus* 120Q. (**f** and **g**) Immunoblot and quantification. Expression of 120Q-huntingtin-induced cleaved caspase-12 (36 kDa band) and this was reduced by addition of 0.3 *μ*M PRE084. *β*-actin was used as control. Values represent means±S.D., *n*=3. *P< 0.05 for 120Q *versus* C

We previously reported that expression of N-terminal mutant huntingtin proteins in PC6.3 cells leads to cell death characterized by activation of various caspases.^6, 9^ To study whether the Sig-1R has a role in cell degeneration, we stimulated PC6.3 cells with PRE084 after transfection of cells with N-terminal mutant huntingtin proteins. Data showed that PRE084 protected PC6.3 cells against mutant huntingtin-induced cell degeneration as shown by estimation of cell viability (Figure 2a) and counting the number of apoptotic nuclei (Figure 2b). PRE084 also counteracted caspase-3 cleavage (17 kDa fragment) that was induced by mutant huntingtin proteins (Figures 2c and d), and reduced the cleavage of PARP that is a target for the active caspase-3 (Figure 2c). Using a commercial kit, we observed that the expression of 120Q-huntingtin induced caspase-3 activity that could be blocked by PRE084 (Figure 2e). Previous studies have shown that procaspase-12, which resides in the ER is cleaved in cells expressing the 120Q-huntingtin-fragment proteins.^6, 9^ PRE084 also prevented the cleavage of caspase-12 in 120Q-huntingtin-expressing cells (Figures 2f and g), suggesting a cytoprotective effect of PRE084 related to reduced ER stress. To study whether the increase in cell viability inversely correlate with mutant huntingtin aggregates, we treated 120Q-huntingtin-expressing cells with PRE084 for 24 h. Data showed that the amount of mutant huntingtin aggregates was not altered by PRE084 treatments nor did the number of cells with aggregates change under these conditions (Supplementary Figure 2).

### Mitochondrial Ca^2+^ levels are not affected by mutant huntingtin proteins or by PRE084 stimulations

**Figure 3:**
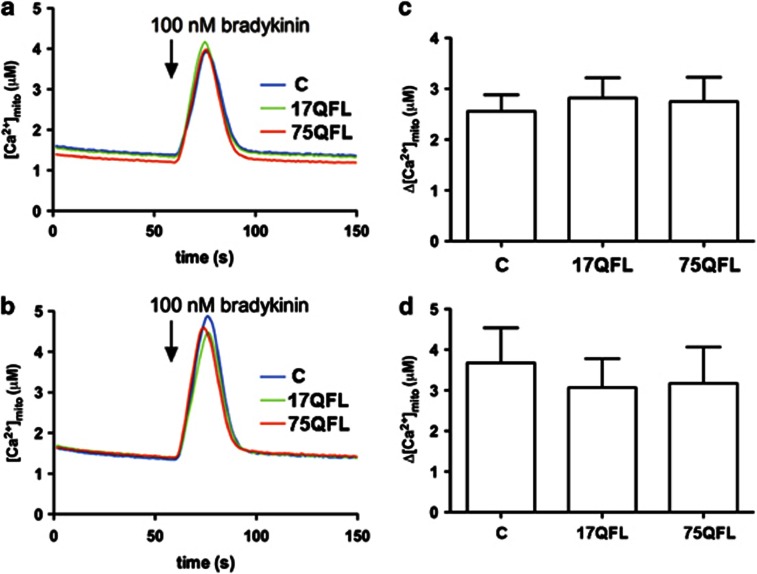
No effects of huntingtin proteins and PRE084 on mitochondrial calcium responses. Mitochondrial Ca^2+^ concentrations were measured in PRE084-treated and in huntingtin protein-overexpressing cells as described in the Materials and Methods. (**a** and **b**) Representative traces of mitochondrial Ca^2+^ concentrations ([Ca^2+^]_mito_) in PC6.3 cells expressing FL huntingtin constructs having 17 (17QFL) and 75 polyglutamine (75QFL) repeats and employing stimulations with 100 nM bradykinin. C, control plasmid pcDNA-expressing cells. Panel **a** is without pretreatment and panel **b** is pretreatment for 20 h with 0.3 *μ*M PRE084. There were no significant changes in [Ca^2+^]_mito._ between controls and PRE084-pretreated cells or after expressing of the mutant huntingtin proteins. (**c** and **d**) Summary histograms. Values are means±S.D., *n*=4. There was a tendency to higher [Ca^2+^]_mito_ following PRE084 treatment but these were not statistically significant from controls

Previous data shows that mutant huntingtin can modulate calcium signaling in neuronal cells through an interaction with IP3R1,^18^ and hat IP3R1 is negatively regulated in a mouse model for HD.^19^ Sig-1R is enriched within the MAM region in the ER^13^ and influences Ca^2+^ signaling from ER into mitochondria by stabilizing IP3R. Given the regulation of Sig-1R by mutant huntingtin (Figure 1), we were interested to study whether the mutant huntingtin proteins may also influence mitochondrial [Ca^2+^] ([Ca^2+^]_mito_) during IP_3_-induced calcium release and whether PRE084 may affect this. To accomplish this, cells were transfected with aequorin targeted to the mitochondrial matrix in conjunction with plasmids expressing either the full-length 17QFL huntingtin or the disease-causing 75Q huntingtin protein plasmids. Control cells were co-transfected with the vector plasmid. The cells were left untreated or pretreated with 0.3 *μ*M PRE084 for 20 h followed by stimulation with 100 nM bradykinin, and the [Ca^2+^]_mito_ was then measured as described in the Materials and methods. Data showed that neither 17QFL nor 75QFL huntingtin altered [Ca^2+^]_mito_ in the cells when compared with untreated controls (Figures 3a and c). Incubation with PRE084 led to a slight increased [Ca^2+^]_mito_ response but this was not significant and [Ca^2+^]_mito_ was not changed in cells expressing 17QFL or 75QFL huntingtin compared with the control (Figures 3b and d). Taken together, the results show that the mutant huntingtin proteins did not influence mitochondrial [Ca^2+^] levels in neuronal PC6.3 cells. In keeping with our previous data using120Q mutant fragment huntingtin protein,^9^ the expression of 75QFL also did not elevate cytosolic Ca^2+^ in the PC6.3 neuronal cells (Supplementary Figure 3).

### Stimulation with PRE084 increases cellular antioxidants and reduces ROS in mutant huntingtin-expressing cells

**Figure 4:**
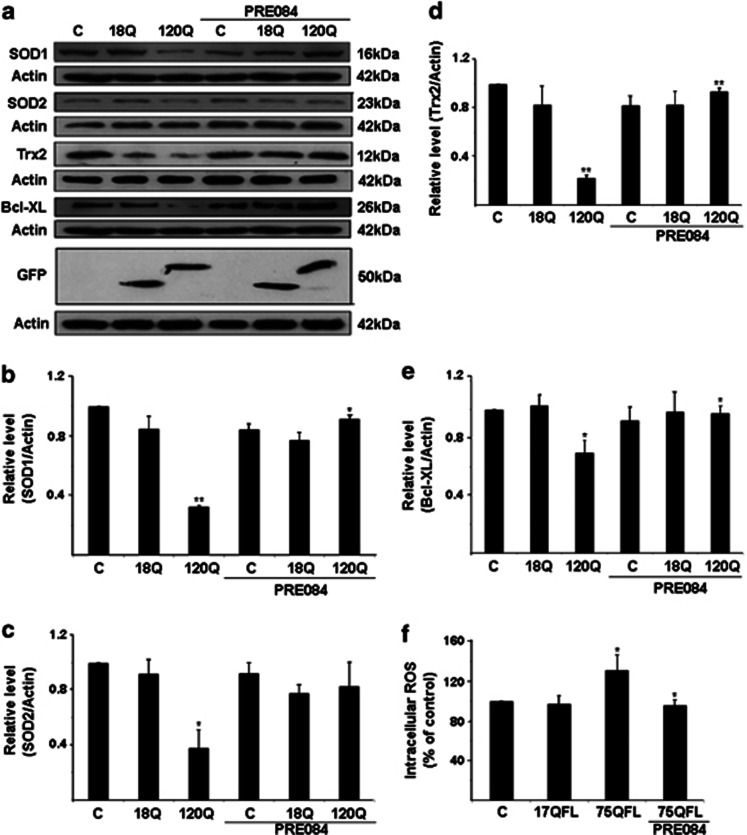
PRE084 increases cellular antioxidants and reduces free radical production in cells expressing mutant *N*-terminal fragment huntingtin proteins. (**a**) Immunoblots were made using specific antibodies as described in the Materials and Methods. Sod1, Sod2, Trx2 and Bcl-X_L_ were decreased in cells expressing 120Q-huntingtin-fragment proteins but the levels were increased by PRE084. *β*-actin was used as control. Expression of the GFP-huntingtin constructs is shown in the lowermost panel. (**b**–**e**) Quantification. Values represent mean±S.D., *n*=3. *P<0.05 or **P<0.01 for 120Q *versus* C and for PRE084+120Q *versus* 120Q. (**f**) Intracellular ROS levels were measured as described in the Materials and Methods. ROS increased in cells expressing the 75QFL huntingtin protein, and this was reduced by 0.3 *μ*M PRE084. Data were normalized to ROS levels in control cells set 100%. Values are means±S.D., *n*=4. *P<0.05 for 75QL *versus* C and for PRE084+75QL *versus* 75QL

We have previously shown that the mitochondrial antioxidant, Sod2 and Trx2, and the cytosolic antioxidants, Sod1 and catalase, are downregulated by the N-terminal mutant huntingtin proteins in PC6.3 cells through downregulation of NF-*κ*B signaling.^9^ The decreased levels of antioxidants were also accompanied by increased production of ROS in cells expressing mutant huntingtin.^9^ To study whether the cytoprotective effect of Sig-1R involves antioxidants, we stimulated cells expressing 120-huntingtin fragment protein with PRE084. Compiling of the data showed that PRE084 was able to increase the cellular levels of various antioxidants that were downregulated by the 120Q-huntingtin (Figures 4a–e). Along this line, stimulation of PC6.3 cells with PRE084 reduced intracellular ROS levels that were elevated by the disease-causing 75QFL huntingtin protein (Figure 4f). These results show that PRE084 is able to decrease the levels of ROS and oxidative stress in neuronal PC6.3 cells expressing mutant huntingtin proteins via the upregulation of cellular antioxidants.

### PRE084 increases NF-*κ*B-p65 levels and activates NF-*κ*B signaling in neuronal PC6.3 cells

**Figure 5:**
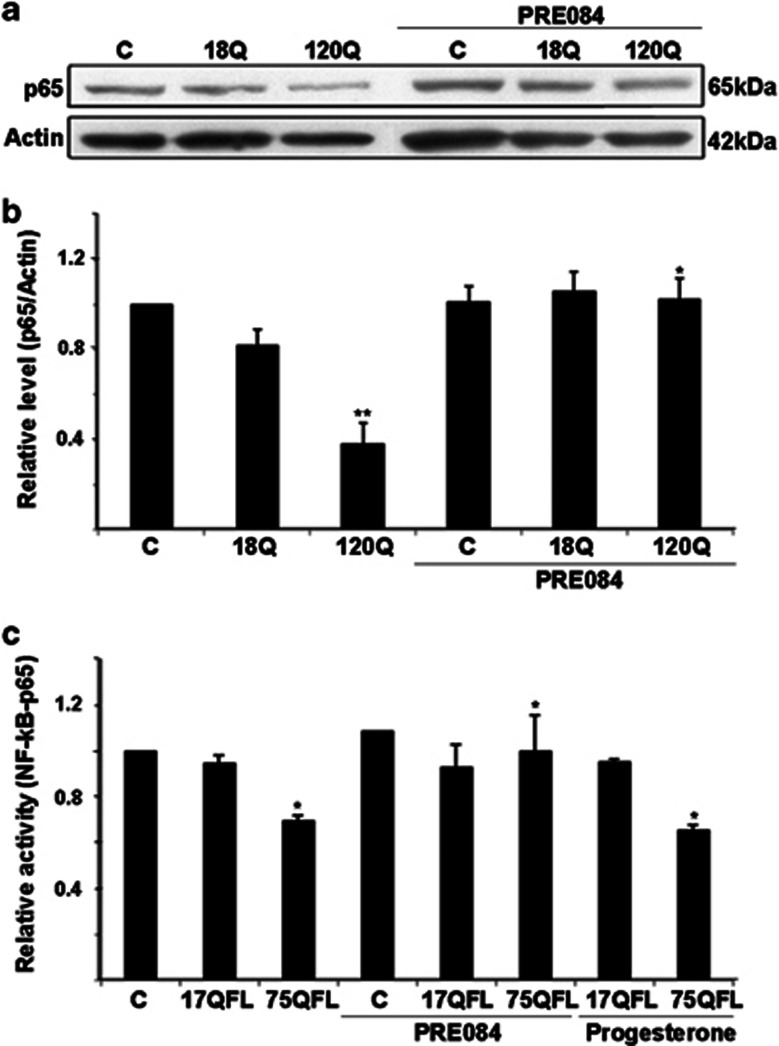
Stimulation with PRE084 counteracts the downregulation of NF-*κ*B pathway induced by mutant huntingtin proteins. (**a** and **b**) Immunoblot and quantification. The levels of NF-*κ*B-p65 were downregulated by expression of 120Q-huntingtin and restored by 0.3 *μ*M PRE084. *β*-actin was used as control. Values represent mean±S.D., *n*=3. **P<0.01 for 120Q *versus* C and *P<0.0 for PRE084+120Q *versus* 120Q. (**c**) NF-*κ*B reporter assay using the luciferase reporter plasmid was performed as described in the Materials and methods. NF-*κ*B activity was decreased in cells expressing FL mutant huntingtin protein (75QFL) and this was restored by addition of 0.3 *μ*M PRE084. Progesterone stimulating also Sigma-2 receptors did not have the same effect. Values represent mean±S.D., *n*=3. *P<0.05 for 75QL *versus* C and for PRE084+75QFL *versus* 75QFL as well as for Progesteron+75QFL *versus* C

To study the involvement of NF-*κ*B signaling in the antioxidant effects of Sig-1R in PC6.3 cells, we determined the levels of NF-*κ*B-p65 (p65, RelA) protein that is important for NF-*κ*B signaling.^20^ Treatment of the cells with PRE084 was able to restore the NF-*κ*B-p65 levels that were downregulated by the expression of 120Q-huntingtin-fragment protein (Figures 5a and b). Similar data was obtained using the disease-causing 75QFL huntingtin and PRE084 (data not shown). Using a NF-*κ*B-luciferase reporter construct, it was also found that the NF-*κ*B activity was decreased in cells expressing 75QFL huntingtin, whereas the 17QFL huntingtin had no effect (Figure 5d). Most importantly, treatment with PRE084 prevented the decrease in NF-*κ*B activity in 75QFL huntingtin-expressing cells (Figure 5c). In contrast, progesterone that acts in part also via the Sigma-2 receptor did not have any effect on NF-*κ*B activity (Figure 5c).

### PRE084 and overexpression of Sig-1R elevate calpastatin in mutant huntingtin-expressing cells

Previously it was shown that calpastatin is decreased in cells expressing the 120Q-huntingtin-fragment protein.^9^ The activity of calpain was also elevated in the 120Q-huntingtin-expressing cells in line with the function of calpastatin as an inhibitor of calpain.^9^ We observed that the mRNA levels of calpains are proteolytic enzymes activated by increased cell calcium and are involved in the regulation of oxidative stress and other processes in the cell.

**Figure 6:**
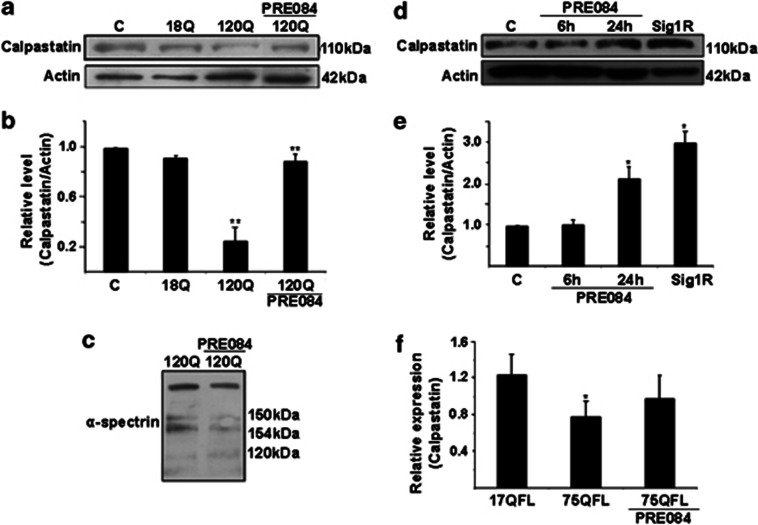
Sig-1 receptors and its agonist, PRE084 upregulate calpastatin in PC6.3 cells. (**a** and **b**) Immunoblot and quantification. Calpastatin level was decreased in cells expressing 120Q-huntingtin and was restored by using 0.3 *μ*M PRE084. *β*-actin was used as control. Values represent mean±S.D., *n*=3. **P<0.01 for 120Q *versus* C. and for 120Q-PRE084 *versus* 120Q. (**c**) Immunoblot. Expression of 120Q-huntingtin induced the appearance of the calpain-specific 145-kDa cleavage product of *α*-spectrin and this was reduced by 0.3 *μ*M PRE084. Typical blot is shown and was repeated three times. (**d** and **e**) Immunoblot and quantification. Stimulation of cells for 24 h with 0.3*μ*M PRE084 increased calpastatin levels compared with control (lanes 1–3). Overexpression of Sig1-R also increased calpastatin levels (lane 4). Values represent mean±S.D., *n*=3. *P<0.05 for PRE084 *versus* C and for Sig1-R *versus* C. (**f**) Calpastatin mRNA levels were determined in cells expressing either the 17QFL or the 75QFK huntingtin protein by quantitative PCR using specific primers for calpastatin as described in the Materials and Methods. The mutant 75QFL huntingtin decreased calpastatin mRNA and this was not significantly counteracted by PRE084. Values are means±S.D., *n*=3. *P<0.05 for 75QFL *versus* 17QFL

We observed here that stimulation of the PC6.3 cells for 6 h with PRE084 increased the calpastatin levels that are reduced by overexpressing 120Q-huntingtin (Figures 6a and b). The increase in calpastatin by PRE084 was accompanied by a reduced calpain activity as shown by decreased cleavage of the calpain substrate, *α*-spectrin in the PC6.3 cells (Figure 6c). To study whether PRE084 elevate calpastatin in control cells, we stimulated cells with the compound for up to 24 h. Data showed that calpastatin levels were significantly induced by PRE084 at 24 h and that overexpression of Sig-1R in the PC6.3 cells had the same effect (Figures 6d and e). To reveal whether calpastatin mRNA levels were also increased by PRE084, we performed qPCR that showed no change in calpastatin expression following PRE084 (Figure 6f). This suggests that the Sig-1R may primarily influence the stability or the degradation of calpastain in cells expressing mutant huntingtin proteins.

## Discussion

The present study shows that the modulation of Sig-1R in neuronal PC6.3 cells can protect against cell degeneration induced by mutant huntingtin proteins. The beneficial effect of the Sig-1R agonist PRE084 was observed both in cells expressing the 120Q-huntingtin-fragment protein and the disease-causing 75QFL huntingtin protein. The mechanism by which PRE084 induces neuroprotection was ascribed to the restoration of the calpastatin and NF-*κ*B-p65 levels in huntingtin-expressing cells, which then upregulated various cellular antioxidants and decreased ROS levels with a positive effect on cell survival. We have previously shown that inhibitors of calpain activities as well as calpastatin overexpression increase PC6.3 cell survival and counteract the deleterious effects caused by mutant huntingtin proteins.^9^ This together with the present results demonstrate that the calpastatin/calpain system is an important regulator of NF-*κ*B-p65 in neuronal PC6.3 cells and that the NF-*κ*B signaling is adversely influenced by the mutant huntingtin proteins. Previous studies have further shown that the I*κ*B kinase *β* (IKK*β*), which is involved in NF-*κ*B signaling can contribute to the neurotoxicity observed with mutant huntingtin.^21^ Here we present evidence that the Sig-1R agonist PRE084 can positively affect the NF-*κ*B pathway and induce neuroprotection of cells expressing mutant huntingtin.^22^ In view of the robust inhibition of cell death and various caspases observed in the PC6.3 cells by PRE084 (Figure 2), this compound may have also other targets in the cells that add to its cytoprotection including effects on anti-apoptotic genes such as *Bcl-2*.^14, 21^

Previously it was shown that the levels of Sig-1R are reduced in various neurodegenerative diseases such as AD and PD.^15, 16^ The mechanisms involved and the functional significance of these changes, however, are not fully understood. Recently, a mutation in Sig-1R was found in patients with a juvenile form of ALS,^17^ and PRE084 was shown to promote motoneuron survival in an animal model of ALS.^23^ We show here that the Sig-1R is decreased in neuronal PC6.3 after expression of mutant huntingtin proteins and can be restored by the agonist PRE084. In cells, Sig-1R is largely localized in the ER in the MAM sub-compartment, which links it close to the mitochondria. We observed a partial co-localization of Sig-1R with aggregates in mutant huntingtin protein-expressing cells, suggesting that the Sig-1R may be redistributed or delocalized in these cells. The agonist PRE084 is thought to stabilize Sig-1R and may therefore hinder its relocalization to protein aggregates containing mutant huntingtin proteins. However, so far this remains a possibility only, and more studies are required to reveal the precise mechanisms by which PRE084 affects the cellular functions of Sig-1R in neuroprotection. Here we analyzed whether PRE084 may influence the mitochondrial [Ca^2+^] levels, which is based upon the observations that Sig-1R is present in MAM and stabilizes the IP3R1.^13^ Furthermore, mutant huntingtin can interact with ER^6, 9^ and modulate calcium signaling in HD.^18, 19^ Using the mitochondrial calcium reporter aequorin, we show here that neither PRE084 nor expression of mutant huntingtin proteins influenced [Ca^2+^]_mito_ levels in the PC6.3 cells (Figure 3). This is an important observation, as deregulation of mitochondrial [Ca^2+^] may in turn lead to an increased ROS production by this organelle with deleterious functional consequences.^24^ PRE084 did not directly affect the [Ca^2+^]_mito_ in mutant huntingtin-expressing cells compared with controls. However, PRE084 did have an effect on the mitochondria-localized antioxidants, Sod2 and Trx2 by increasing their levels in 120Q-huntingtin-expressing cells (Figure 4). The increased levels of Sod2 and Trx2 probably contribute to the decrease in ROS levels observed with PRE084. We have previously shown that Sod2 and Trx2 in cells are regulated by NF-*κ*B signaling.^9, 25^ The activation of NF-*κ*B by PRE084 may underlie the beneficial effects of this compound in oxidative stress. Apart from the dysregulated NF-*κ*B signaling, there is evidence for an inhibition of cytoprotective autophagy in the PC6.3 cells expressing mutant huntingtin proteins.^26^ This in turn may increase the amount of protein aggregates enhancing cell stress and further disrupt protective signals in the cells leading to a *circulus vitiosus* scenario. It is therefore important to try to target the more upstream events in the cellular cascades leading to the disease.

Since the identification of the Sig-1R-binding sites in 1982, several selective Sig-1R ligands have been synthesized.^27, 28, 29, 30, 31^ Pharmacological studies have shown that such ligands may have many physiological effects ranging from neuroprotection to neuropsychiatric and anti-depressant effects.^10, 11, 32^ Of the many compounds interacting with these receptors, the selective Sig-1R agonists PRE084 (ref. 28)) and 1-(3,4-dimethoxyphenethyl)-4-(3-phenylpropyl) piperazine dihydrochloride (SA4503)^29, 33^ have been mostly studied. PRE084 have been shown to have beneficial effects in various models of brain diseases including neurodegenerative and acute brain disorders. Thus, PRE084 was shown to promote cell survival and reduce oxidative stress caused by ischemia^34^ and toxicity induced by *β*-amyloid peptide.^35^ PRE084 was also neuroprotective against nerve avulsion injury^36^ and it acts on motoneurons both *in vivo* and *in vitro*.^23, 37^ Here we show that PRE084 counteracts the deleterious effects of mutant huntingtin proteins in neuronal PC6-3 cells by increasing calpastatin and by activating NF-*κ*B signaling. Moreover the Sig-1R was reduced in mutant huntingtin proteins expressing cells and could be restored by PRE084 treatments. It has previously been shown that calpain is abnormally activated in striatal regions of HD patients and in animal models of HD.^38, 39^ In this context, the present data showing that PRE084 can increase calpastatin expression and thereby control calpain activity can be of functional significance in HD. It remains to be studied whether Sig-1R is altered in HD and whether PRE084 or other Sig-1R ligands may exert beneficial effects in animal models of HD. Taken together, the results in this study have identified a signaling pathway mediating neuroprotection in cells expressing mutant huntingtin involving the Sig-1R and the calpastatin/NF-*κ*B signaling pathway. The data indicate that compounds influencing Sig-1R may constitute promising targets for future drug developments in HD.

## Materials and Methods

### Cell culture and transfections

PC6.3 neuronal cells were cultured in RPMI-1600 (Lonza, Basel, Switzerland) medium supplemented with 5% fetal calf serum (Chemicon, Billerica, MA, USA) and 10% horse serum. Cells were transfected with expression vectors encoding different CAG-repeat lengths of huntingtin exon-1 fused to EGFP and FL huntingtin constructs with 17- and 75-polyglutamine repeats, as described earlier.^6, 9^ Transfections were done using the Transfectin reagent (BioRad, Hercules, CA, USA) with the above plasmids or with the control EGFP expression plasmid (Clontech, Mountain View, CA, USA). 0,3 μM PRE084 (Tocris, Bristol, UK) and 3 μM progesterone (Sigma-Aldrich, St. Louis, MO, USA) were added 4 h after transfection.

Cell viability was determined by the MTT [3-(4,5-dimethylthiazol-2-yl)-2,5-diphenyltetrazolium bromide (Calbiochem) assay as described previously.^9, 40^ Hoechst 33342 (Sigma) was employed to stain dying cells showing condensed and fragmented DNA.^39, 40^ More than 300 fluorescent cells in each well were analyzed and experiments were repeated three times. Results are expressed as percentage of transfected cells.

### Immunocytochemistry

PC6.3 cells plated on poly-lysine and laminin-coated coverslips were fixed for 7 min using 4% paraformaldehyde. Cells were incubated for 1 h using phosphate-based saline (PBS) containing 0.1% Triton-X-100 and 5% bovine serum albumin (Sigma), followed by incubation overnight with primary Sig-1R (1 : 200; Abcam, Cambridge, UK) and EM48 (1 : 300, Millipore, Billerica, MA, USA). Cells were washed using PBS and incubated for 1 h using Alexa594-conjugated secondary antibodies (1 : 300, Molecular Probes, Invitrogen, Carlsbad, CA, USA). Cells were counterstained for 1 min using Hoechst 33342 blue (4 *μ*g/ml; Sigma), mounted in gel mounting medium (DABCO) and analyzed using a Zeiss LSM confocal microscope (Zeiss, Oberkochen, Germany) at the Helsinki Biomedicum Molecular Imaging unit. Controls without primary antibodies showed no staining.

### Immunoblots

Cells were lysed in RIPA buffer (150 mM NaCl, 1% Triton-X-100, 0.5% sodium deoxycholate, 50 mM Tris-HCl and 0.1% SDS pH 8.0) containing phosphatase inhibitor and protease inhibitor cocktail (Roche, Basel, Schweiz) and protein concentrations were determined by Supersignal West pico (Thermo Scientific, Rockford, IL, USA). Equal amount of protein (40 μg) were separated on SDS-PAGE and proteins were blotted onto a nitrocellulose filter (Amersham Biosciences, Piscataway, NJ, USA). Filters were blocked for 1 h in 5% milk-TBS or 5% BSA-TBS followed by an overnight incubation at 4 °C using primary antibodies.^41, 42^ These included antibodies against Sig-1R (1 : 1000; Santa Cruz Biotechnology, Santa Cruz, CA, USA) NF-*κ*B-p65 (1 : 250; Santa Cruz Biotechnology), SOD1 (1 : 10 000, Santa Cruz Biotechnology), SOD2 (1 : 15 000, LabFrontier, Seoul, Korea), GFP (1 : 5000; Roche), Trx2 (1 : 1000; LabFrontier), calpastatin (1 : 1000; Santa Cruz Biotechnology), Bcl-X_L_ (1 : 1500, BD Transduction Laboratories, San Diego, CA, USA), active caspase-12 (1 : 1000, Chemicon), active caspase-3 (1 : 350, Cell Signaling Technology), PARP (1 : 1000, Cell Signaling Technology), calpastatin (1 : 1000 Santa Cruz Biotechnology), *α*-spectrin (1 : 1000, Chemicon), and *β*-actin (1 : 3000; Sigma). After washing, the filters were incubated with horseradish peroxidase-conjugated secondary antibodies (1 : 2500, Pierce, Rockford, IL, USA), followed by detection using the enhanced chemiluminescent method (Pierce).

### Solubility assay to detect mutant huntingtin aggregates

Cells were lysed in buffer containing 50 mM Tris-HCl, pH 7.5, 100 mM NaCl, 3 mM EGTA, 0.5% Triton-X and protease inhibitors (Roche) and kept for 5 min on ice and suspended in 3 volumes of SDS loading buffer to obtain the total cell lysate as described previously.^43^ Aggregation of huntingtin fragment proteins was observed by blotting the stacking gel to reveal high-molecular-weight protein species.^6^

### Caspase assay

PC6.3 cells were transfected with 18Q- and 120Q-expressing plasmids as noted above and 0.3 μM PRE084 was added 4 h after transfection as indicated. Controls were transfected with EGFP expression plasmid. Cells were incubated for 2 days and caspase-3/7 activities were measured using a Caspase-Glo assay kit (Promega) as described by the vendor. Samples were incubated at room temperature for 90 min and the luminescence was measured using a luminometer (GloMax 20/20, Promega, Biofellows, Helsinki, Finland).

### Reverse transcription and quantitative PCR

Total RNA was extracted from cells using the GenElute Mammalian total RNA kit (Sigma) and gene-specific reverse transcription was performed using Tetro reverse transcriptase (Bioline, London, UK). cDNA synthesis was carried out in a 10-*μ*l reaction volume containing 1 × Tetro reaction buffer, 0.5 *μ*M each of the reverse primers (*calpastatin*, 5′-CCCCAGTAGACTTCTCTTTC-3′ *Sig1R,* 5′-CTTCCTCTACATTCCTCTG-3′), 750 ng of the RNA template and 0.5 *μ*l Tetro reverse transcriptase. The reaction was incubated at 75 °C for 5 min to open RNA secondary structures, and samples were cooled to 42 °C. Superscript III reverse transcriptase was then added and the samples were incubated at 42 °C for 20 min followed by enzyme inactivation for 20 min at 64 °C. The unbound reverse transcription primers were digested with 20 units Exonuclease I (New England Biolabs GmbH, Frankfurt-Hoechst, Germany) at 37 °C for 30 min, followed by denaturation at 80 °C for 15 min.

One microliter of product was then amplified with 1 × Light Cycler 480 SYBR Green I Master Mix (Roche Diagnostics GmbH, Mannheim, Germany) and using 0.5 *μ*M of forward (fw) and reverse (rev) primers (*calpastatin*, fw: 5′-AGTAGTTCTGGACCCAATG-3′, rev: 5′-CCCCAGTAGACTTCTCTTTC-3′ *Sig1R*, fw: 5′-TGCCTTATCTCCATTCCA-3′, rev: 5′-CTCCTTCCTTCAGTCCTT-3′). qPCR amplification and relative quantifications were performed on the Light Cycler 480 II instrument (Roche) using 40 cycles (10 s denaturation at 95 °C, 20 s annealing at 60 °C and 20 s extension at 72 °C) and essentially as described.^44^ Melting curve acquisition and analysis was also carried out immediately after amplification to confirm the specificity of PCR.

### Measurement of intracellular ROS

PC6.3 cells were cultured in 96-well plate and transfected with FL huntingtin constructs with 17- and 75 polyglutamine repeats for 48 h, 0.3 μM PRE084 was added 4 h after transfection. Cells were treated with 10 μM 6-carboxy-2′,7′-dichlorodihydrofluorescein diacetate, di(acetoxymethyl ester) (H_2_DCFDA AM: Invitrogen, Carlsbad, CA, USA) diluted in PBS buffer and incubated at 37 °C for 45 min. H_2_DCFDA is able to penetrate cells due to the acetoxymethyl ester, where it is hydrolyzed by intracellular esterases to form 2′,7′-dichloro-fluorescin (DCFH). Oxidation of DCFH by hydrogen peroxide and hydroxyl radicals yields a highly fluorescent product 2′,7′-dichlorofluorescein (DCF). The fluorescence intensity of DCF after excitation of the samples at a wavelength of 485 nm was measured at an emission wavelength of 535 nm using a fluorescence microplate reader. Results are shown as normalized to control (set 100%).

### Aequorin-based measurements of Ca^2+^ concentration in mitochondria

Measurements of mitochondrial Ca^2+^ concentration ([Ca^2+^]_mito_) were carried out using recombinant aequorin targeted to mitochondrial matrix (mtAeq) and a luminometer system as described previously.^45, 46^ In brief, cells were grown to 70% confluence on poly-ℒ-lysine-coated 13-mm glass coverslips and transfected with the mtAeq (kind gift from Professor U Ruegg, Geneva, Switzerland) along with control (pcDNA), 17QFL- or 75QFL-huntingtin-expressing plasmids as indicated in Figure 3. At 24 h after transfection, the cells were washed with HBSS buffer (118 mM NaCl, 4.6 mM KCL, 1 mM CaCl_2_, 10 mM 𝒟-glucose, 20 mM HEPES, pH 7,4) and reconstituted with 5 *μ*M native coelenterazine (Invitrogen, CA, USA) for 1 h at room temperature. Cells were then placed into a perfusion chamber and the luminescence was recorded. All measurements were conducted in HBSS at 37°C. Cells were stimulated with 100 nM bradykinin (Sigma-Aldrich, MO, USA) to induce the release of Ca^2+^ from the intracellular stores. Calibration of the measurements was done by permeabilizing the cells in HBSS containing 10 mM Ca^2+^ and 100 *μ*M digitonin (Sigma-Aldrich), thus generating the maximal luminescence of the sample. Luminescence data was then converted to [Ca^2+^] according to Brini *et al.*^46^

### Measurement of intracellular calcium

Cells were grown on poly-ℒ-lysine-coated coverslips and transfected for 24 h as described above. The cells were then washed three times with HBSS buffer (118 mM NaCl, 4.6 mM KCl, 1 mM CaCl_2_, 10 mM 𝒟-glucose, 20 mM HEPES, pH 7.4), following a 30-min incubation with 2 *μ*M Fura-2 AM at room temperature. After washing twice using HBSS, the cells were transferred to a heated coverslip holder adjusted to 37 °C, and perfused with HBSS containing 100 nM bradykinin. An XBO 75W/2 xenon lamp served as the source for excitation light and the excitation filters were set at 340 or 380 nm, respectively. Emitted light was measured at 510 nm. Filters were controlled with Lambda 10-2 (Sutter Instruments, Novato, CA, USA). A SensiCam CCD camera was employed for image acquisition and the recorded images were processed using the Axon Imaging Workbench software (Axon Instruments, Foster City, CA, USA). The F340/F380 fluorescence ratio served as the indication of intracellular calcium concentrations.

### NF-*κ*B reporter assay

PC6.3 cells in six-well plates were transfected with 0.5 *μ*g of FL huntingtin expression plasmids in conjunction with 0.5 *μ*g of the NF-*κ*B reporter plasmid, containing multiple NF-*κ*B sites linked to firefly *luciferase* gene. A volume of 0.02 *μ*g of the Renilla luciferase pRL-TK was used as control for transfection efficiency. Cells were harvested 48 h after transfection using Passive Lysis Buffer. Renilla and firefly luciferase activities were measured using the dual luciferase substrate and a luminometer (GloMax 20/20).^25, 47^ Results are shown as fold increase in luciferase normalized to the Renilla activity.

### Quantification and statistics

Immunoblots were quantified with ImageJ quantification software. Results are expressed as percentage of controls (mean±S.E.M.). Statistical analyses were performed using one-way ANOVA and Bonferroni's multiple comparison tests. Values are given as means±S.E.M. and *P*<0.05 was considered as statistically significant.

## Abbreviations

AD: Alzheimer's disease
ALS: amyotrophic lateral sclerosis
Bip: binding immunoglobulin protein
GRP-78: glucose-regulated protein (78 kDa)
ER: endoplasmic reticulum
HD: Huntington's disease
IP3R: inositol 1,4,5-triphosphate receptor
IRE1: inositol-requiring enzyme 1
MAM: mitochondria-associated membrane
PC6.3: pheochromocytoma cell line subline 6.3
PERK: protein kinase RNA-like endoplasmic reticulum kinase
polyQ: polyglutamine
PRE084: 2-(4-morpholino)ethyl1-phenylcyclohexane-1-carboxylate hydrochloride
qPCR: quantitative polymerase chain reaction
ROS: reactive oxygen species
Sig-1 R: Sigma-1 receptor
UPR: unfolded protein response
