# Supplementary figures and images for: Sigma-1 receptor agonist PRE084 is protective against mutant huntingtin-induced cell degeneration: involvement of calpastatin and the NF-κB pathway
Source: Cell Death Dis. 2013 May 23;4(5):e646–. doi: 10.1038/cddis.2013.170 (PMC3674377; doi:10.1038/cddis.2013.170)

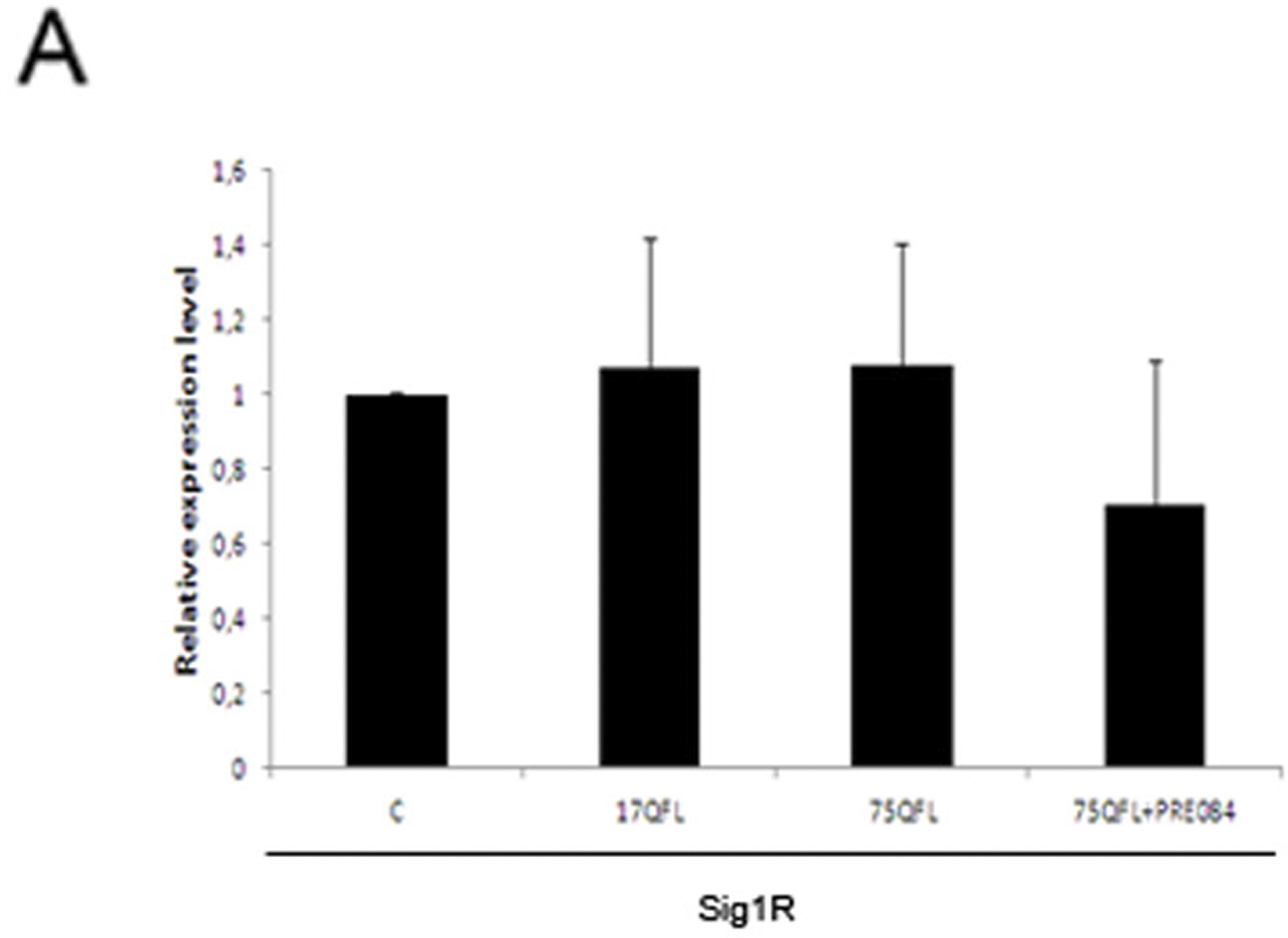

Supplement: Supplementary Figure 1 [file cddis2013170x1.tif]

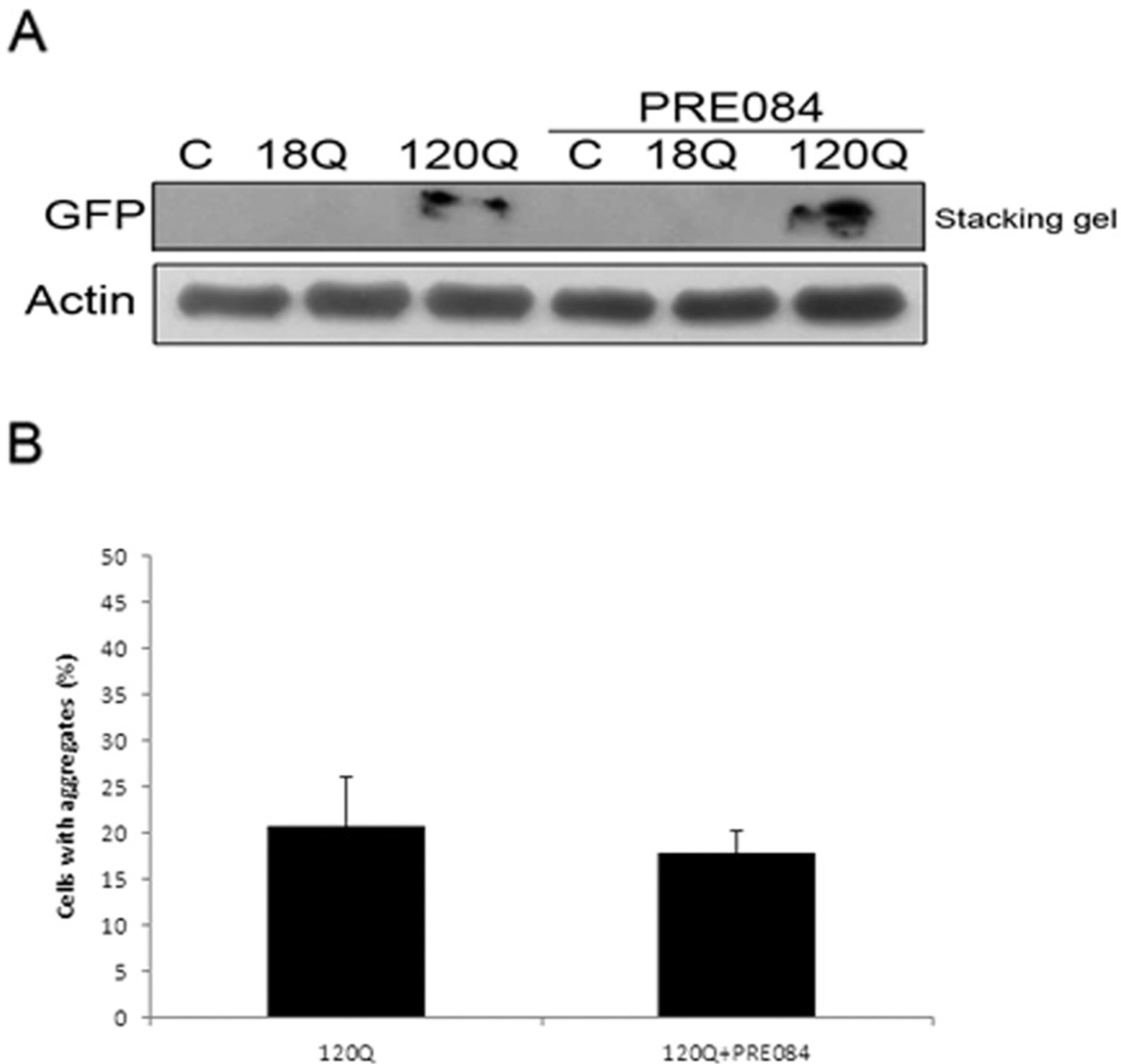

Supplement: Supplementary Figure 2 [file cddis2013170x2.tif]

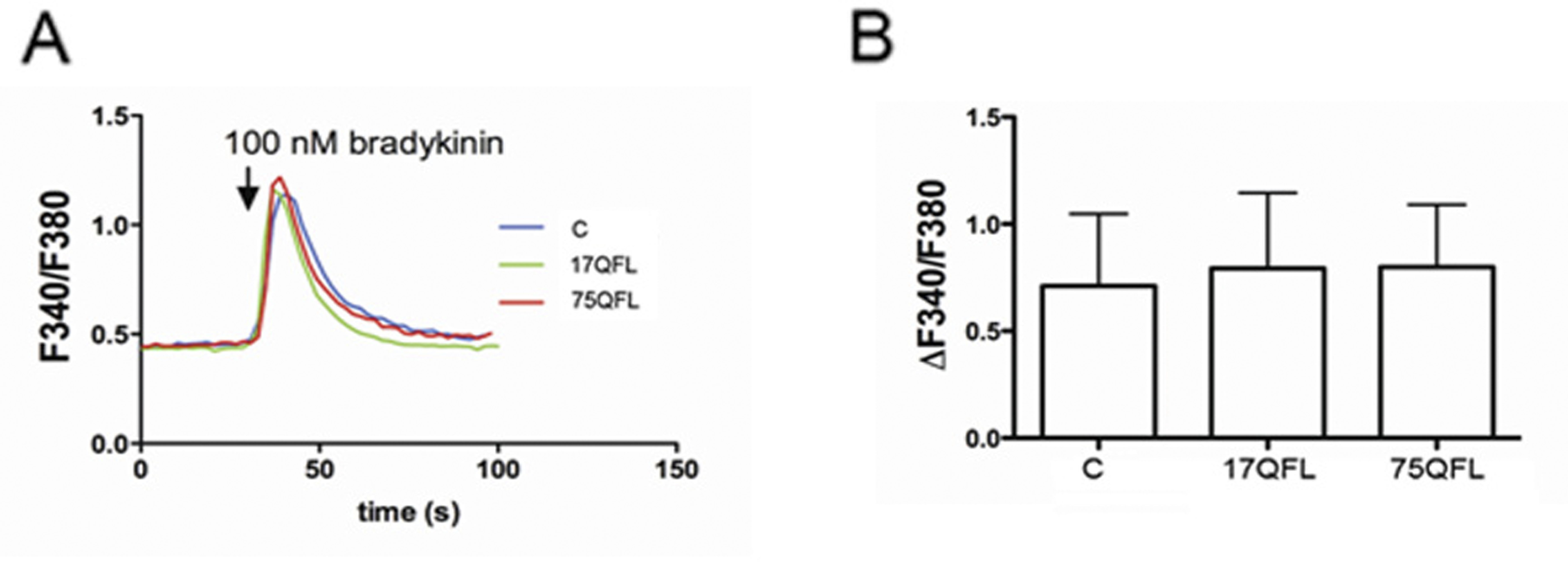

Supplement: Supplementary Figure 3 [file cddis2013170x3.tif]
