# Supplementary material for: Sigma-1 receptor agonist PRE084 is protective against mutant huntingtin-induced cell degeneration: involvement of calpastatin and the NF-κB pathway
Source: Cell Death Dis. 2013 May 23;4(5):e646–. doi: 10.1038/cddis.2013.170 (PMC3674377; doi:10.1038/cddis.2013.170)
Supplement: Supplementary Figure Legend [file cddis2013170x4.doc]

**Supplementary Information**

**Figure Legends**

**Supplementary Figure 1**. **Sig-1R mRNA expression in neuronal PC6.3 cells**

Quantitative PCR was done as described in Methods using specific primers for SigR1. There were no significant changes in Sig-1R mRNA levels between samples.

**Supplementary Figure 2. Effect of PRE084 on aggregates induced by 120Q-huntingtin fragment protein.**

(A) Aggregates of mutant huntingtin were analyzed by immunoblotting in lysates from PC6.3 cells as described in Methods. Note induction of aggregates by 120Q-huntingtin as revealed by anti-GFP antibodies. This was not affected by the treatment with 0.3 µM PRE084. Typical experiment is shown and was repeated three times.

(B) The number of PC6.3 cells with aggregates after 120Q-huntingtin expression was not changed by 0.3 µM PRE084. More than 100 cells were counted in three wells and the experiment repeated three times.

**Supplementary Figure 3.** **Huntingtin expression does not alter bradykinin-induced intracellular calcium transients.**

Intracellular calcium was measured as described in Methods.

(A) Representative traces showing changes in intracellular calcium concentration in PC6.3 cells expressing full-length huntingtin constructs having 17 (17QFL) and 75 polyglutamine (75QFL) repeats after stimulation with 100 nM bradykinin. pcDNA-expressing cells served as controls.

(B) Summary of the results. Values are means ± SD, n=72 individual cells. No significant changes in intracellular calcium transients were observed.
